# Estrogen receptor-α signaling in post-natal mammary development and breast cancers

**DOI:** 10.1007/s00018-021-03860-4

**Published:** 2021-06-22

**Authors:** Mariam Rusidzé, Marine Adlanmérini, Elodie Chantalat, I. Raymond-Letron, Surya Cayre, Jean-François Arnal, Marie-Ange Deugnier, Françoise Lenfant

**Affiliations:** 1 INSERM U1297, Institut Des Maladies Métaboliques et Cardiovasculaires, Université de Toulouse - UPS, CHU, Toulouse, France; 2 LabHPEC et Institut RESTORE, Université de Toulouse, CNRS U-5070, EFS, ENVT, Inserm U1301, Toulouse, France; 3 Department of Cell Biology and Cancer, Institut Curie, PSL Research University, Sorbonne University, CNRS UMR144 Paris, France

**Keywords:** Mammary gland, 17β-estradiol, ERα-positive luminal cells, Lineage specification, Stem cells

## Abstract

17β-estradiol controls post-natal mammary gland development and exerts its effects through Estrogen Receptor ERα, a member of the nuclear receptor family. ERα is also critical for breast cancer progression and remains a central therapeutic target for hormone-dependent breast cancers. In this review, we summarize the current understanding of the complex ERα signaling pathways that involve either classical nuclear “genomic” or membrane “non-genomic” actions and regulate in concert with other hormones the different stages of mammary development. We describe the cellular and molecular features of the luminal cell lineage expressing ERα and provide an overview of the transgenic mouse models impacting ERα signaling, highlighting the pivotal role of ERα in mammary gland morphogenesis and function and its implication in the tumorigenic processes. Finally, we describe the main features of the ERα-positive luminal breast cancers and their modeling in mice.

## Introduction

The mammary gland is an exocrine gland of ectodermal origin whose primary function is to produce milk for the nourishment of offspring. In humans as in most mammals, mammary morphogenesis is initiated during the embryonic period but the most important part of mammary development and remodeling occurs after birth, throughout puberty, pregnancy, lactation and involution [1–6]. Despite some differences, the human and mouse mammary epithelium shares strong similarities in developmental processes, cellular organization and signaling molecules [4, 7]. Mouse models are, therefore, widely used to decipher the molecular mechanisms controlling the development and homeostasis of the mammary gland, and analyze their deregulation upon tumorigenic processes.

The post-natal development of the mammary gland and its function are controlled by a hormonal network that mainly comprises estrogens, progesterone, prolactin, growth hormone (GH) and oxytocin [3, 8]. Prolactin, GH and oxytocin are peptide hormones of pituitary origin, whereas estrogens and progesterone are steroid hormones primarily produced by ovaries during reproductive life. Pioneering works showing that ovariectomized and ERα-deficient mice were unable to develop mammary gland at puberty have indicated that signaling through estrogens is crucial for the post-natal mammary development [9–12]. In addition, ERα is routinely used as a diagnosis marker supporting the molecular classification of breast cancers [13–15] and remains an essential therapeutic target for hormone-dependent breast cancers, in particular through administration of tamoxifen (TAM) and/or aromatase inhibitors (AI), that both are very efficient in reducing the risk of cancer recurrence [16–18].

As member of the nuclear receptor family, ERα has a well-established transcription factor activity and controls the expression a large spectrum of target genes [19, 20]. However, estrogens and ERα can also act at the cell membrane level to induce non-genomic events [21, 22]. The recent development of new transgenic mouse models and omics-based analyses has allowed to better characterize the ERα-positive luminal cell lineage and to further dissect the complex signaling events triggered by estrogens in the mammary epithelium. Here, we review the current understanding of the mechanisms of ERα actions, derived from different studies on mammary development, stem cell function and tumorigenesis.

## ERα and its modes of action

In humans and rodents, two distinct estrogen receptors, ERα and ERβ, have been identified. They show large sequence homology and similar binding affinity for 17β-estradiol (E2), the predominant form of circulating estrogens [19, 23]. *Esr1* (*ESR1* in human) encoding ERα was first identified in 1986 [24, 25] and located on a different chromosome than *Esr2* coding for ERβ, identified later in 1996 [26]. ERα is believed to be the ancestral steroid receptor originating 400–500 million years ago [27] and its complex modes of action and gene organization remain abundantly studied [28]. In vivo, perturbation of ERα signaling has a major impact on mammary development [11, 12], whereas ERβ loss does not result in a deleterious mammary phenotype and impaired function [29, 30].

**Fig. 1:**
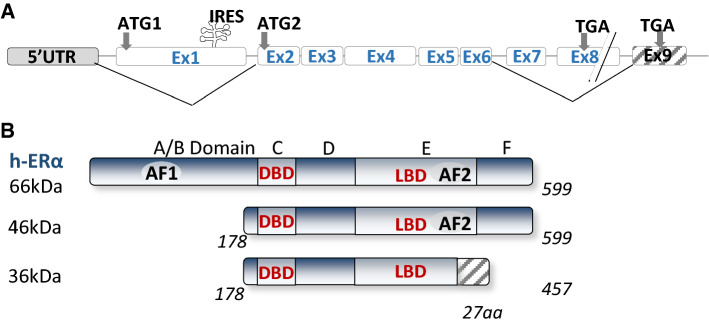
Structure of the *ESR1* gene and the different isoforms of ERα. On the top, the coding exons are annotated following the nomenclature published in [32]. Alternative splicing that generates the shorter ERα46 and ERα36 isoforms are indicated using solid lines

*ESR1* gene spans over 300 kb and consists of nine coding exons and seven introns (Fig. 1). The first eight exons encode the major full-length 66 kDa isoform of ERα [31]. The promoter region (over 150 kb) contains several promoter sequences named A to T that drives its specific expression in target tissues [32, 33]. *ESR1* gene expression is tightly regulated by multiple regulatory elements, including transcription factors, chromatin environment, autocrine, paracrine and endocrine secreted factors, and multiple environment factors (cell–cell and cell–matrix interactions, mechanical forces) [34]. In addition, the 3'UTR region of ERα contains several regulatory elements specific for miRNAs, such as miR18a, miR22, miR206 and miR221/22, that control ERα stability or translocation [35].

ERα is composed of six structural domains namely A to F, including two binding domains, one to DNA (DBD, C domain) and the other to ligand (LBD, E domain) [19, 21]. It also includes a ligand-independent (AF1) and a ligand-dependent (AF-2) subdomain, mapping to the A/B and E domains, respectively [36, 37] (Fig. 1). The AF-1 transactivation domain is mainly ligand independent, its stimulation relying on the phosphorylation of serine 104/106, 118 or 167 by kinases activated downstream of growth factors such as EGF (Epidermal Growth Factor), IGF-1 (Insulin-like Growth Factor-1), or TGFα (Tumor Growth Factor) [38–41]. However, AF-1 can also be modified in response to E2 and further stabilized following phosphorylation on serine 118 [42–44]. The A domain interacts with the C-terminal domain to allow repression in absence of ligand [45]. The D domain is a hinge region that provides flexibility between the DBD and the LBD (E/ F) domains. The mutation of this D region affects the synergy between the AF-1 and AF-2 functions of ERα [46]. AF1 and AF2 display distinct activation functions that are specifically involved in the recruitment of cofactors. These coregulators are not only proteins that link the receptor and the transcription machinery but rather have enzymatic activities that induce chromatin modification and remodeling, and control initiation of transcription [47–49]. Among the coregulators that bind to the AF-2 domain exposed following E2 binding, there are members of the p160 family that includes three analogous factors SRC-1, SRC-2 and SRC-3 (Steroid Receptor Coactivator, part of histone deacetylase) [50, 51]. Other well-known cofactors comprise CBP/p300 and MED1. Interestingly, p160 proteins also interact with the NH2-terminal domain of ERα, in particular the AF1 domain, and p300 allows a functional synergy between AF1 and AF2 [40, 52]. This was confirmed by the recent quaternary structure of an active ERα-coregulator complex on DNA identified using cryoelectron microscopy [53]. Moreover, ERα also interacts with some corepressors, such as the repressor of estrogen receptor activity (REA) repressor which binds on the LBD domain in a ligand-dependent manner [54] or RIP140 (receptor interacting protein) through a direct competition with SCR-1 [55].

### Natural isoforms of ERα

In addition to the “classic” full-length isoform of ERα (ERα-66 kDa) which contains the two AF-1 and AF-2 activation functions, there is a shorter 46 kDa isoform lacking the first 173 amino acids and, therefore, the AF-1 function (Fig. 1). Although the prominent, if any, mechanisms accounting for the expression of the ERα46 isoform still remain to be clarified, three possible processes of generation were reported: (*i*) an alternative splicing that generated a mRNA deficient in the nucleotide sequence corresponding to exon 1 encoding the A/B domain generation [56]; *(ii)* proteolysis [57, 58]; and *(iii)* initiation of translation at a downstream ATG which encodes methionine 174 in the human ERα66 by an IRES (Internal Ribosome Entry Site) located within the full-length mRNA [59]. A recent study showed that the expression of ERα46 is due to the action of the oncoprotein HMGA1a (High Mobility Group A protein1a) that regulates the alternative splicing of *ESR1* in MCF7 breast cancer cells [60]. Overexpression of ERα46 in proliferating MCF7 cells provokes a cell cycle arrest in G0/G1 phases and inhibits the ERα66-mediated estrogenic induction of all AF-1-sensitive reporters: c-fos and cyclin D1 as well as estrogen-responsive element-driven reporters [56, 61]. The role of the AF-1-deficient ERα46 isoform has also been questioned in vivo using a “knock in” strategy. These mice (named *ER*α*AF-1*^*0*^) only express a short 49 kDa isoform that lacks 441 nucleotides from exon 1 and is functionally similar to ERα46 [62]. The females are sterile, with uterine atrophy while they conserved several vasculoprotective actions of E2 [62–64]. Studies on mammary gland development are reported later in chapter 4.1.

Western blot with antibodies directed against the C-terminal domain is the unique procedure to detect the ERα46 isoform since ERα46 and ERα66 share identical aminoacid sequences that cannot be distinguish by immunohistochemistry. Although the ERα46 isoform has not been studied extensively, it was found expressed in various cell types such as vascular endothelial cells and macrophages [65–68]. ERα46 is also expressed in breast cancer cells including tamoxifen-resistant cells [69] and in more than 70% of human breast tumors with highly variable expression levels, sometimes even more abundant than the ERα66 protein [70]. Importantly, higher amounts of ERα46 proteins were associated with highly differentiated tumors of lower grade and smaller size [70].

In 2005, another shorter 36 kDa isoform of ERα was identified from a human endometrium cDNA library [71]. This ERα36 isoform is transcribed from an alternative promoter located in the first intron of the *ESR1* gene and is encoded by exons 1, 2–6, and 9 (Fig. 1). ERα-36 thus lacks the transactivation functions AF-1 and AF-2 but retains the DNA-binding domain of ERα66 and its partial dimerization and ligand-binding domains. It also contains a unique 27 amino acids at the C-terminus that replaced the last 138 aminoacids encoded by exons 7 et 8 and can be detected by specific antibodies. ERα36 contains three potential myristoylation sites which are conserved in the full-length ERα66. These are residues 25–30 (GVWSCE), 76–81 (GMMKGG) and 171–176 (ELLTNL) [71]. Myristoylation being a post-translational modification allowing anchoring to the plasma membrane, ERα-36 was suggested to be mainly localized at the plasma membrane where it could relay rapid estrogen signaling and inhibit the transcriptional activity of ERα 66 kDa, probably by competition at DNA-binding sites [71, 72]. The ERα36 receptor is not expressed in mice. However, it was found largely expressed in both ERα-positive and ERα-negative breast cancers, at a proportion that varies between 40 and 50% according to cohort studies [73–75]. ERα36 is mainly described in the literature to be involved in the acquired resistance to anti-estrogen drugs, such as tamoxifen and in the progression of mammary tumors in response to chemotherapy [76].

### Complexity of actions of ERα signaling

**Fig. 2:**
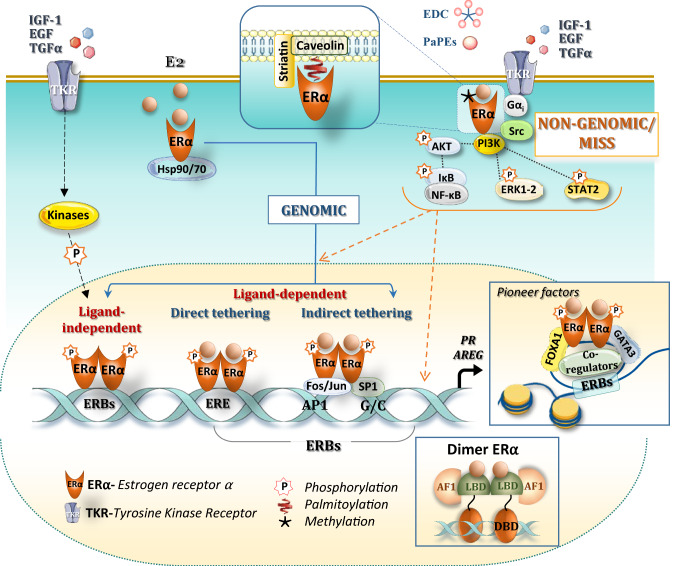
Estrogen receptor ERα signaling. Classic ERα signaling leads to genomic actions through ligand-receptor binding, leading to dimerization of ERα that binds directly to specific DNA sites (called estrogen response elements, ERE) that activate transcription. ERα can also bind by indirect tethering to other transcription factors, such as AP1 or SP1 (blue line). The ERα can also be activated in a ligand-independent manner through downstream events of receptor tyrosine kinases (RTKs) activated by growth factors in the mammary gland, such as IGF-1, EGF (blue dotted line, in particular through phosphorylation of serine residues in the AF-1 domain). Induction of transcriptional response depends on the chromatin remodeling, induced by pioneer factors such as FoxA1 and GATA-3 in the mammary gland, and is modulated by the specific recruitment of coregulators. Non-genomic, membrane-initiated steroid signaling (MISS) actions involve a small pool of ERα located on the extracellular compartment or close to the membrane, at least in part through direct interaction with caveolin-1 in response to post-translational modifications such as palmitoylation. Transient methylation of arginine 260 has also been observed to induce ERα interaction with the p85 subunit of PI3K and Src, Upon E2 binding, these non-genomic activations activate the subsequent interaction of ERα with protein kinases (Src and PI3K), G-coupled protein I, leading to activation of signaling cascades (Akt, ERK1/2) and further shuttle of these phosphorylated transcription factors in the nucleus. These non-genomic signaling pathways are rapidly activated and further induce genomic activations (orange dotted line)

ERα activation is a complex process involving many signaling pathways that trigger either classical nuclear “genomic” or membrane “non-genomic” actions (Fig. 2).

### The nuclear actions of ERα

As a member of the nuclear receptor family, ERα mainly functions as a ligand-activated transcription factor through different mechanisms (Fig. 2). Estrogen binding to the LBD induces dissociation from the Hsp90/Hsp70-multi-protein chaperone machinery, receptor dimerization and nuclear entry. Crystal structure revealed that the LBD has 12 alpha helices and E2-binding repositionnes helix 12, such that activation function AF-2 is exposed, allowing interactions with coregulators [77]. ERα is then stabilized in its active state and binds directly to specific DNA sites to estrogen-response elements (ERE = 5’GGTCAnnnTGACC3’ palindromic sequences) [78].

About 25% of estrogen-regulated genes lack complete ERE sequences in their promoter regions [79]. Moreover, ERα can bind to DNA by indirect tethering to other transcription factors such as the Stimulating protein 1 (SP1) on sites rich in GC, the jun/c-fos proteins which form a dimeric complex binding to “Activator Protein 1” (AP-1) sites [80] and Nuclear factor–κβ (NF-κβ). Genome-wide analysis of ERα DNA-binding sites has identified not only rigorously dissociate the genomic and, but also PITX1 whose binding motif was found present in 28% of genome-wide ERα-binding sites [81–83].

Studies using CHIP-Chip and CHIP-seq on MCF7 breast cancer cells have revealed that ERα binds to 5000–10,000 locations [84–86]. However, only < 5% of these ERα binding sites (ERBs) are located in the proximal region of ERα target genes and conserved in the mouse genome [79, 87]. Most of these ERBs are distally located from targets genes and function as distal-*cis-*regulatory elements, generating a complex numbers of loops and anchors to bring the receptor binding sites closer to the transcription initiation site [85, 88]. CHIP-seq in the mouse mammary gland identified close to 6000 high confidence ERBs, with half of them enriched in ERE, PAX2, SF1 and AP1 motifs located at distal enhancer regions [89].

Transcriptional activity can also be regulated in a ligand-independent manner through downstream events of receptor tyrosine kinases (RTKs) activated by growth factors such as EGF, IGF-1 or TGFα [38]. Although ligand independent, these effects can be blocked by an anti-estrogen [90, 91]. This can affect either AF-1 on serine residues via phosphorylation by cyclin/Cdk2, MAPK or GSK3, thereby modulating ligand-independent activation of ERα, or AF-2, in particular on Y537 where ligand binding is located [38, 92]. These modifications were shown to be particularly essential for the genomic effects of ERα, in particular for the recruitment of transcriptional co-activators [93–96]. Thus, phosphorylation integrates these signaling pathways, such as epidermal growth factor receptor (EGFR)/human epidermal growth factor receptor 2 (HER2) into a complex cross-talk network with estrogen signaling. [92, 97].

The first cistrome of ERα has been performed in 2006 [84] and allowed to identify close to ERBs, some pioneer factors bound to DNA, in particular FOXA1 “ForkHead Box A1” [98], FOXM1 “ForkHead Box M1” [99], raising the idea that these pioneer factors control accessibility of ERα on chromatin [100]. The same goes for the PBX1 factor [101], and for the factor GATA3 “GATA Binding Protein” [102]. The crucial role of these pioneer factors for the ERα response was demonstrated when FOXA1 and AP2gamma binding to several sites is decreased upon ERα silencing [103] (see also Chapter 4.2 for their roles in the mammary gland development).

### The membrane “non-genomic” actions of ERα

A small fraction of the ERα is found at the plasma membrane where it activates the so-called “rapid”, “non-genomic”, or MISS for “Membrane-Initiated Steroid Signaling”, which induces multiple signaling pathways [49, 104] and creates cross-talk between membrane and nuclear signaling [21, 22] (Fig. 2). The first rapid effect was described in 1967 when AMPc production was found to be increased within minutes in response to 17β-estradiol in the uterus [105]. The hypothesis of receptors, localized to the plasma membrane was then emitted but was controversial until 1977, when E2 binding was observed in membrane isolated from endometrial cells and hepatocytes [106]. Meanwhile, high number of data has shown that E2 rapidly activates G proteins, and a number of kinases such PI3K, P21ras, c-Src/ERK1-2 [21, 107]. Membrane ERα has near identical affinity for E2 than nuclear ERα and originates from the same transcript, but its abundance is very low (around 3% as compared to nuclear ERα) [108]. The so-called membrane ERα is localized within lipid rafts called caveolae within the plasma membrane, and S522A mutant of ERα was 60% less effective than wt ERα in binding caveolin-1 [109]. The receptor will thus form a real signaling platform made up of several proteins such as caveolin, striatin, Src, G proteins or even growth factors. Striatin directly binds to amino acids 183–253 of ERα, targets ERα to the cell membrane, and serves as a scaffold for the formation of an ERα-G_αi_ complex [110]. Post-translational modifications, such as palmitoylation on Cys 447 (451 in mice) allows membrane anchoring by its palmitate [111, 112] and the membrane-initiated signaling (MISS). Transient methylation of arginine 260 has also been observed to induce ERα interaction with the p85 subunit of PI3K and Src, recruiting also the focal adhesion kinase (FAK) in this complex [113].

Membrane ERα effects were studied using transgenic mouse models mutated either for the palmitoylation site (ERα-C451A, murine counterpart of human C447) [114, 115], or the methylation site (R264A, murine counterpart of human R260) [116]. Rapid signaling was also blocked by overexpression of a peptide that prevents ERs from interacting with the scaffold protein striatin (the disrupting mouse peptide) [117]. This membrane localization is crucial on endothelial cells where membrane ERα are coupled to eNOS in a functional signaling module that may regulate rapid NO synthesis and acceleration of re-endothelialization by E2 (reviewed in [21]).

To rigorously dissociate the genomic and non-genomic activities of E2, John Katzenellenbogen has developed two pharmacological tools to specifically activate the rapid membrane signaling: (i) the Estrogen-dendrimer conjugate (EDC), which can cross the plasma membrane but cannot enter the nucleus due to its charge and size [118] and (ii) the “pathway preferential estrogens” (PaPEs) which only activate non-genomic signaling, due to their very low affinities and rapid dissociation rates [119]. About 25% of genes responding to E2 also respond to EDC in MCF7 cells [120]. In contrast, the specific inhibition of PI3K, MAPK or even c-Src kinases by chemical inhibitors lead to a significant deregulation of the transcriptional response induced by E2, demonstrating that estrogen signaling interacts with other pathways allowing the establishment of a complete transcriptional response [120]. The integration of non-genomic actions of E2 at the chromatin level was also perfectly illustrated by the work of Miguel Beato's laboratory. Five minutes after hormone treatment, the cytoplasmic signaling cascade Src/Ras/Erk is activated via an interaction of the progesterone receptor with ERα leading to chromatin remodeling and cell proliferation [121].

In view of these studies, it is, therefore, difficult to functionally dissociate these two actions of estrogenic signaling. It is conceivable that, according to the cell type, differentiation and environment, genomic and membrane-initiated signaling (MISS) can act i) in concert, participating synergistically in the transcriptional initiation of hormone receptors in general, through post-translational modifications and epigenetic modifications of the chromatin, or ii) independently following the concept of moonlighting proteins [122, 123], playing one role in the extranuclear compartment, as already demonstrated in the endothelium, and one genomic, transcriptional role in the nuclear compartment.

## Mammary development and cell lineages

### Overview of the post-natal mammary development and its hormonal context

**Fig. 3:**
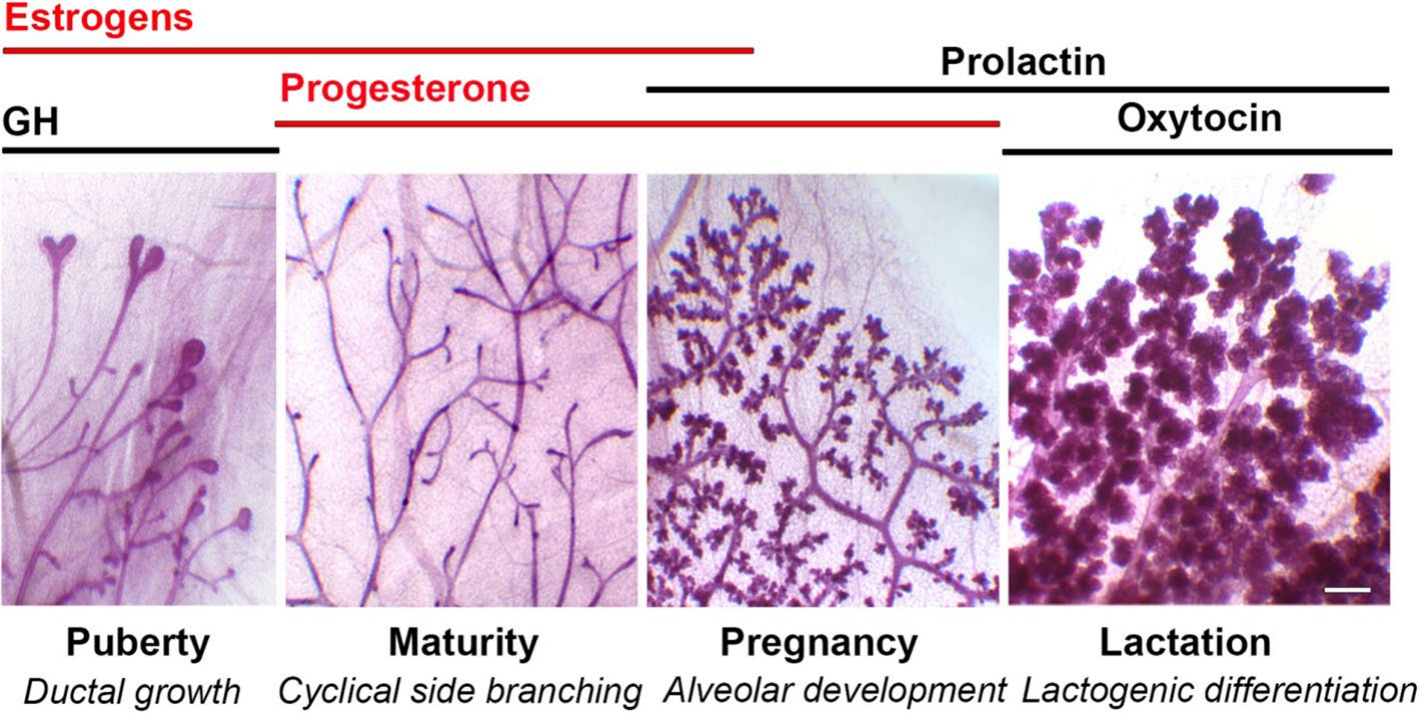
Hormonal context of the major stages of the post-natal mouse mammary development. From left to right: images of carmine-stained whole mounts from 6-week-old pubertal, 12-week-old adult virgin, 16-day-pregnant and 2-day lactating mice. The pubescent gland is characterized by the presence of terminal end buds (TEBs) at the tips of the growing ducts. The steroid hormones, estrogens and progesterone, are in red whereas the peptide hormones are in black. *GH* growth hormone. Bar: 0.25 mm

Comprehensive reviews on mammary development have been recently published [4–6]. An overview of each mammary developmental stage and their different hormonal contexts is provided below and illustrated in Fig. 3.

**Fig. 4:**
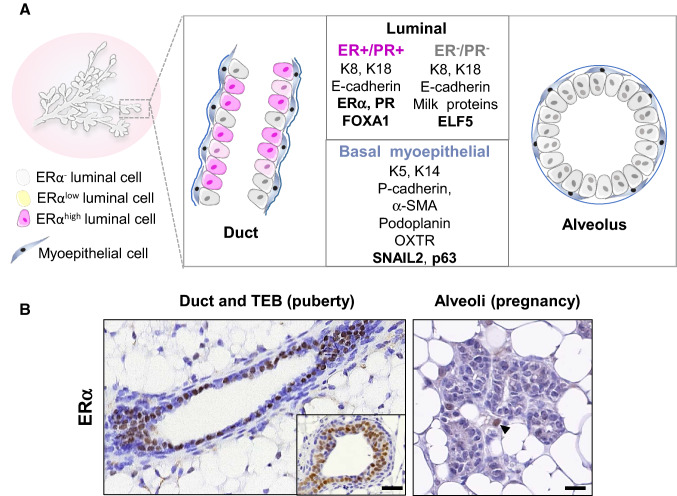
Organization of the mammary bilayer and localization of ERα-expressing cells. **A** Schematic representation of mammary duct and alveolus and main specific markers of the basal myoepithelial, ERα-positive and –negative luminal cell lineages. Ductal ERα^high^ cells express nuclear ERα as detected by IHC whereas ERα^low^ cells express ERα transcripts without detectable nuclear staining. Keratins (K5, K14, K8, K18); α-smooth muscle actin (α-SMA); oxytocin receptor (OXTR). **B**: ERα expression in ductal and alveolar structures, as revealed by IHC on PFA-fixed paraffin sections, using mouse monoclonal anti-ERα (Santa Cruz, sc-542, MC-20, described in [212]). Left: sections through a duct and a TEB (insert) from a pubertal mouse. Right: section through a group of alveoli from a pregnant mouse. Unlike ductal, alveolar luminal cells rarely display ERα nuclear expression. The arrowhead points to an ERα cell located in the stroma. Bars, 50 μm and 25 μm

The mammary gland consists of a ramified epithelial tree embedded into a fatty stroma. When fully differentiated, the mammary tree is composed of milk-secreting alveoli connected by branching ducts (Fig. 3). In ducts and alveoli, the mammary epithelium is composed of an inner layer of luminal cells lining a lumen and an outer layer of basal myoepithelial cells sitting on a basement membrane (Fig. 4A). During lactation, the secretory luminal cells produce milk components, whereas the contractile myoepithelial cells serve for milk expulsion [1, 124, 125].

In mouse females, the mammary tree remains rudimentary until puberty. At about 4 weeks of age, initiation of puberty triggers ductal elongation and branching, a process associated with elevated levels of GH and 17β-estradiol (E2), [2, 8, 126] (Fig. 3). Cell proliferation mainly occurs at the tips of growing ducts within specialized bulbous structures, the terminal end buds (TEBs), that are composed of several inner layers of luminal-type cells and an outer layer of basal-type cells, known as body and cap cells, respectively [127]. TEBs drive ductal progression through the fat pad, with coordinated cell proliferation, differentiation, apoptosis and migration events [128–130]. At sexual maturity, TEBs regress and ductal elongation ceases. Estrogen and progesterone levels fluctuate with the recurrent estrus cycles, peaking during the pre-ovulatory (proestrus and estrus) and post-ovulatory (diestrus) phases, respectively [126, 131]. Cell proliferation and apoptosis successively occur with each cycle, leading to the formation of side branches and nascent alveolar buds that wax at the diestrus stage and partially regress thereafter [126, 131, 132].

During pregnancy, the mammary secretory tissue undergoes a massive expansion and prepares for milk production. Alveolar buds are formed all over the ductal tree and progressively develop into secretory alveoli that will be mature and fully functional upon lactation [1]. These processes are accompanied by an early surge of estrogen followed by a peak of progesterone. Concomitantly, levels of prolactin increase [126, 131]. Around parturition, progesterone levels abruptly drop down resulting in induction of labor. Prolactin levels remain high throughout lactation together with oxytocin, a hormone that controls myoepithelial cell contractility and milk ejection [1, 125, 133]. At weaning, the secretory tissue goes through a controlled process of cell death leading to involution and the gland returns to a pre-pregnant-like state until a novel cycle of gestation and lactation [6].

Ovariectomy of prepubertal females impedes mammary development, whereas administration of exogeneous estrogens restores its growth, resulting in morphological changes similar to those observed at puberty [9, 134]. Noticeably, the response of the mammary tissue to estrogen stimulation is dose dependent. Low-to-moderate doses induce TEB formation and ductal elongation, while these processes are inhibited at higher doses [135]. This dose response effects underline the complex action of estrogen signaling on mammary gland and may have a physiological significance since E2 levels are lower during the pubertal growth than during pregnancy.

Thus, elevated levels of circulating estrogens are associated with two major morphogenetic events, the pubertal ductal growth and the onset of alveolar expansion at gestation. Of note, signaling of mammotropic hormones synergizes at multiple levels. In particular, estrogens induce the expression of progesterone receptor (PR) and prolactin receptor (PRLR) transcripts, highlighting the pivotal role of ERα signaling in the hormonal response of the developing mammary epithelium [136–138].

### Mammary basal and luminal lineages

It is now established that stem cells drive the post-natal mammary development. Pioneering orthotopic transplantation studies have shown that basal cells isolated from the adult mammary epithelium were able to regenerate bilayered ducts and alveoli, even at single cell level, whereas luminal cells had no significant regenerative potential [139–141]. This observation, confirmed by numerous subsequent transplantation assays, initially supported the notion that basal-type multipotent stem cells generated the myoepithelial and luminal cell lineages during puberty and pregnancy [4].

However, recent data from lineage-tracing experiments revealed that in situ, the post-natal mammary development and its homeostasis are essentially sustained by distinct basal and luminal unipotent stem cells [142–146]. Two distinct luminal lineages have been identified, relying on the presence or absence of ERα expression [144, 145] (Fig. 4A). The ERα-positive lineage is viewed as a hormone-sensing entity acting through paracrine mechanisms on basal and luminal ERα-negative cells, whereas the ERα-negative lineage is largely committed to milk secretion [3, 131, 147]. Interestingly, recent data have shown that under regenerative conditions and upon oncogene expression, adult basal cells can reactivate a multipotency program that is restricted in situ by luminal cells through secretion of tumor necrosis factor [148].

## Characteristics of the ERα luminal cell lineage

### Distribution of ERα ^+^ luminal cells within the developing mammary epithelium

Immunohistochemical (IHC) studies have shown that ERα is expressed in the nuclei of both mammary epithelial and stromal cells [9]. The presence of epithelial but not stromal ERα turned to be essential for mammary morphogenesis [12].

Throughout development, nuclear ERα is absent from the basal myoepithelial cells and confined to the luminal layer. Interestingly, the proportion of ERα^+^ cells in the luminal compartment varies according to the developmental stage of the gland [145, 149–151]. Absent at birth, ERα was detected in about half of luminal cells at post-natal day 7, a proportion maintained during the pubertal growth [138, 145]. During puberty, ERα is present in ductal luminal cells and in luminal body cells of TEBs [149, 150] (Fig. 4B). In post-pubertal virgin mice, ducts still comprise at least 50% of ERα^+^ luminal cells. This percentage decreases to about 5% at the end of pregnancy, the remaining positive cells being primarily located in ducts. During lactation, the luminal layer of the functional alveoli consists of ERα negative secretory cells [145, 150] (Fig. 4A).

Interestingly, detection of ERα transcripts in situ using RNAscope indicated that the status of ERα expression in luminal cells seems more complex than that observed by IHC [138]. This approach highlighted the existence of three luminal subsets in the mammary epithelium of pubertal females: 20% of luminal cells were found negative for both ERα mRNA and protein, 40% positive for ERα mRNA but negative for the protein (termed ERα^low^) and 40% positive for both ERα mRNA and protein (termed ERα^high^). Whether ERα^high^ and ERα^low^ cells represent mature and progenitor cells or reflect a continuous gradient in ERα expression levels remains to be determined. Another open question is whether ERα^low^ cells express membrane ERα and constitute a particular subset of estrogen-sensing cells. Indeed, in endothelial cells, ERα acts at the cell membrane level but cannot be visualized in the nucleus by immunostaining [21].

### ERα ^+^ luminal cells as hormone-sensing cells

*PGR* is an established estrogen-target gene encoding the nuclear receptor isoforms, PR-A and PR-B [131, 152, 153]. Both isoforms (hereafter referred to as PR) have been detected in the mouse mammary epithelium, but only PR-B is required for a proper mammary development [154, 155].

Consistent with the role of estrogens in inducing *PGR*, most luminal cells staining positive for ERα^ +^ by IHC display a nuclear expression of PR, in mouse as well as in human mammary epithelium [156–158]. Moreover, the luminal cells from ERα knock-out mice completely lack nuclear PR expression [138, 159–161]. Interestingly, analysis of transgenic mice lacking either AF-1 or AF-2 domain of ERα revealed that PR expression in mammary luminal cells is primarily AF-2 dependent, i.e., ligand-dependent [138]. This study also indicated that PR is preferentially expressed by the ERα^high^ luminal subset.

Mammary luminal cells co-expressing ERα and PR (ERα ^+^ PR^ +^) are perceived as the main targets of the ovarian steroid hormones and consequently are termed hormone-receptor positive, hormone-sensing or sensor cells [4, 131, 162]. Of note, several gene expression profiles of mouse mammary epithelial cells have shown that the luminal cell population expressing *Esr1 and Pgr* also contain high levels of *Prlr* transcripts, indicating that it responds to prolactin stimulation, in addition to estrogens and progesterone [7, 162–166]. However, the lack of reliable antibodies against PRLR has hampered the precise localization of this receptor in situ.

### Enrichment of ERα ^+^ luminal cells by flow cytometry

The use of a panel of cell surface markers for flow cytometry has allowed a clear separation of the mammary basal, luminal and stromal cell populations and, in addition, enabled the enrichment the ERα^ +^ PR^ +^ and ERα^-^PR^-^ luminal cell subsets. The most commonly used markers are summarized in Table 1. A large fraction of them are adhesion molecules, such as EpCAM, CD24, ICAM-1 and the integrin chains α2, α6, β1 and β3.

**Table 1:** Major surface markers used to separate mouse mammary basal and luminal cells and enrich ERα-positive and ERα-negative luminal cell populations by flow cytometry after exclusion of endothelial and hemopoietic cells by CD31 and CD45 surface staining

Surface marker	Basal	Luminal ERα^+^	Luminal ERα^−^	References
Used to separate basal from luminal cells				
CD24	+	+ +	+ +	[173]
EpCAM	+	+ +	+ +	[164]
CD29 (β1-Itg)	+ +	+	+	[139]
CD49f (α6-Itg)	+ +	+	+	[140]
Podoplanin	+ +	–	–	[167]
Used to enrich ERα^+^ and ERα^−^ luminal cells				
CD61 (β3-Itg)	+ +	–	+	[169]
CD49b (α2-Itg)	+ +	–	+	[164]
c-Kit	–	–	+	[171]
CD14	–	–	+	[163]
ICAM-1	+ +	–	+	[172]
Sca-1	–	+	–	[141]
CD133 (Prominin-1)	–	+	–	[141]

In the human mammary epithelium, ERα ^+ ^PR ^+^ luminal cells are characterized by a lower α6 (CD49f) integrin expression than ERα^- ^PR^-^ cells and CD49f separates quite nicely the two populations [168].

In mouse, ERα^+^PR^+^ luminal cells have been enriched using differential expression of Sca-1, Prominin-1 (CD133), c-Kit, CD14, ICAM-1 and the α2- (CD49b) and β3 (CD61) integrin chains [141, 163, 164, 169–172] (Table 1). None of these markers perfectly discriminate the two luminal populations. However, some of them (Sca-1, Prominin-1 and ICAM-1) display a robust expression in various mouse genetic backgrounds, at protein and mRNA levels [141, 162, 163, 166, 172, 174, 175].

Most ERα ^+ ^PR^ +^ luminal cells are positive for Sca-1 and Prominin-1 (Table 1). So that the use of these markers enables a convenient enrichment of the hormone-sensing population [141, 144, 163, 175]. This was confirmed by purifying the ERα-positive lineage following its tracing by YFP expressed under the control of ERα promoter [145]. Although ICAM-1 and CD49b largely mark the ERα^-^PR^-^ luminal cell population (Table 1), they are expressed by a minor subset positive for Sca-1 and enriched in ERα^+^PR^+^ cells. This Sca-1^ +^ ICAM-1 ^+^ (or Sca-1 ^+ ^CD49b^ +^) subset has a colony-forming potential, attributed to ERα^ +^ luminal progenitors [164, 172].

Mammary gland function and ERα expression are not altered in Prominin-1 knockout females, suggesting that this glycoprotein is not an essential regulator of the ERα ^+ ^PR ^+^ lineage [176]. Similarly, loss of ICAM-1 and CD49b does not result in a deleterious mammary phenotype [42, 172].

### Main molecular features of ERα ^+^ luminal cells

Global transcriptomic profiles of the ERα^ +^ PR^ +^ and ERα^- ^PR^-^ luminal cell populations enriched by flow cytometry have been established in human and mouse mammary tissues [7, 163, 164, 166, 174]. More recently, comprehensive and unbiased gene expression analyses across different stages of mammary development were performed using single cell RNA-seq [162, 177–179]. Apart from *Esr1* and *Pgr*, a few genes encoding transcription factors and coregulators (*Foxa1*, *Tbx3, Msx2*, *Myb* and *Cited1*) have been reported as specifically expressed in the hormone-sensing cell population of the adult gland [7, 162–164]. Of note, the hormone-sensing cell population is devoid of *Elf5*, a key transcription factor controlling the alveolar cell fate [7, 144, 163, 166, 180]. *Elf5* specifically signs the ERα^- ^PR- luminal cell subset together with milk protein genes, such as β-casein and WAP (whey acidic protein).

In agreement with the gene expression data, IHC studies have shown that the vast majority of ERα^ +^ luminal cells co-expresses FOXA1, an inducer of ERα expression that controls its transcriptional activity [98, 150, 181]. Consistently, GATA3, a transcription factor regulating both ERα and FOXA1 mRNA expression, is present in ERα ^+^ luminal cells [158, 182–184]. A correlation was also observed between the presence of ERα and TBX3, a transcriptional repressor involved in the generation of the hormone-sensing cell population [185]. CITED1, a transcriptional coactivator of ERα, has been detected in a subset of ductal and TEB luminal cells (most probably ERα^+^) during puberty [181, 186].

Transcriptomic profiles and gene expression analysis by qRT-PCR also revealed that the ERα^+^PR^+^cell population highly expresses several genes encoding secreted factors, such as *Areg* (encoding amphiregulin)*, Tnfsf11* (encoding RANKL) and *Wnt4* [7, 162, 163, 166]. WNT4 and RANKL are effectors of progesterone signaling. They play a major role in ductal side branching and alveologenesis during pregnancy by inducing the expansion of basal and ERα^- ^PR^-^ luminal cells through paracrine mechanisms downstream of PR activation [152, 187–189].

*Areg* is an established ERα-target gene strongly induced in the mammary glands of ovariectomized females stimulated by E2 and restricted to luminal cells expressing *Esr1* and *Pgr,* as seen by single-cell RNA-seq analysis [190, 191]. *Areg* transcripts have been detected in situ in a subset of luminal cells expressing PR that most probably belong to the ERα^high^ population [138, 190]. The role of AREG downstream of ERα signaling in the mammary epithelium will be described in the section dedicated to the transgenic mouse models.

A recent single cell RNA-seq analysis comparing mammary glands from young (3–4 month-old) and aged (13–14 month-old) virgin mice revealed age-dependent alterations in cell type composition and gene expression that potentially reflect age-associated hormonal changes [192]. The proportion of hormone-sensing cells decreases with age and their transcriptomic profile is characterized by the up-regulated expression of Tph1 (encoding tryptophan hydroxylase 1) and Arg1 (encoding arginase 1). In line with previous works [162, 177], this study also identified a rare luminal population that co-expressed hormone-sensing and secretory-alveolar lineage specific genes, suggesting a dual differentiation potential. Transcriptional data were further supported by in situ detection of luminal cells co-expressing ERα, PR and milk-related markers (MFGE8, LTF). Notably, the abundance of this population whose precise in vivo function remains to be determined strongly decreases with aging.

### ERα ^+^ unipotent stem cells

Early observations on tissue sections revealed that unlike ER^- ^PR^-^ luminal cells, ER^ +^ PR ^+^ cells rarely display proliferation markers [156, 161]. Hormone-sensing cells were, therefore, for long time, considered as mature luminal cells with poor growth ability. In addition, it has been suggested that ERα expression is lost before the proliferative response, as stimulation with E2 led to undetectable ERα expression within 4 h that reappeared by 24 h [134]. Nonetheless, later on, several studies using nucleotide analog incorporation assays indicated that ERα ^+ ^PR ^+ ^luminal cells substantially proliferate in particular during puberty, during the estrus stage and at the beginning of gestation [149, 151, 174, 193]. Moreover, in vitro colony-formation assays on isolated cells showed that although less clonogenic than ERα^- ^PR^-^ cells, the ERα^ +^ PR^ +^ luminal cell subset defined by the double expression of Sca-1 and ICAM-1 (or CD49b) harbored colony-forming cells, considered as expanding progenitors [141, 164, 172].

Two recent studies using Prominin-1 or ERα expression to map ERα ^+^ cell fate in situ confirmed that ERα^ + ^luminal cells expand during mammary development and in addition showed that these cells exclusively generate an ERα ^+﻿ ^luminal progeny [145, 175]. Specifically, by tracing ERα-positive luminal cells with YFP in transgenic mice, Van Keymeulen et al. reported that lineage-restricted ERα ^+^ luminal stem/progenitor cells ensure the expansion of ERα ^+ ^luminal cells during puberty and sustain their long-term renewal during repeated cycles of pregnancy and lactation [145]. It is not clear yet whether the construct selected in this study allowed the tracing of both ERα^high^ and ERα^low^ cells or preferentially that of ERα^high^ cells, which would impact the interpretation of the data.

ERα ^+ ^luminal stem/progenitor cells remain to be fully characterized. RNA-seq analysis of single mammary epithelial cells isolated from adult mouse indicated that high levels of *Aldh1a3*, *Lypd3*, *Kit* and *Cd14* could discriminate ERα + luminal stem/progenitor cells from their differentiated progeny [162]. However, these genes are also highly expressed in ERα-negative luminal stem/progenitor cells. The analysis of three independent RNA-seq data sets suggests the existence of a common ALDH1A3 ^+ ^ERα^-^ luminal stem/progenitor cell for both ERα ^+ ^and ERα^-^ cell lineages [162, 178, 179].

The molecular mechanisms and signals from the niche that control the ERα^ +^ luminal cell lineage remains to be explored in detail. Notch1 has been identified as a master determinant of the mammary luminal cell differentiation. Its activation represses the basal-specific transcription factor ∆Np63 [194] and can reprogram basal cells into ERα-negative luminal cells in vivo [195]. Notably, ERα and Notch1 expression in post-natal luminal cells is mutually exclusive [144], suggesting a negative cross-talk between Notch and ERα signaling. Consistently, former studies performed with breast cancer cell lines showed that stimulation by E2 inhibited Notch1 activity [196].

A recent work reports that R-spondin 1 (RSPO1), a niche factor secreted by the ERα-negative luminal cells, regulates ERα expression through paracrine mechanisms [197]. RSPO1 is known to bind LGR receptors and synergize with WNT4 to enhance Wnt/βcat signaling in mammary basal cells. Using a luminal cell-specific *Rspo1*-deficient transgenic mouse model, the authors found that loss of RSPO1 resulted in reduced mammary side branching in adult virgin females, with a decreased ERα expression and signaling activity in luminal cells. RSPO1 activated G-protein coupled cAMP signaling in ERα^ +^ luminal cells through LGR4, independently of the Wnt/βcat axis.

## Mammary phenotype of transgenic mouse models impacting ERα signaling

### Mouse models mutated for ERα

The transgenic mouse models used to dissect the role of ERα signaling in mammary development and function are presented in Table 2.

**Table 2:** Transgenic mouse models used to dissect the role of ERα signaling in mammary development

Mouse model	Hormonal levels	Genetic feature	Mammary phenotype	Références
Mouse models mutated for ERα and ERβ				
ERα-Neo-KO	Elevated E2 levels (8X)	Insertion of neomycin resistance cassette into ESR1 exon 1 resulting in an ERα mutant form lacking the functional AF-1	Absence of pubertal development	[11, 198]
ERα- KO	Elevated E2 levels (8X)	Deletion of Exon 2 of Esr1 gene resulting in complete detectable expression of ERα transcript	Absence of pubertal development in the KO females Impaired ductal and alveolar development after transplantation into WT stroma. Epithelial and not stromal ERα required	[12, 190, 199]
MMTV-Cre-ERα ^fl/fl^		MMTV-Cre mice bred with ERα ^fl/fl^ mice to mediated epithelial -specific ablation of ERα gene in virgin mice	Impaired ductal development and side branching in adult virgin females	[201]
WAP-Cre-ERα^fl/fl^		WAP-Cre bred with ERα ^fl/fl^ to mediated epithelial -specific ablation of ERα gene in mice during late pregnancy and lactation	Defective alveolar development and lactation upon successive pregnancies	[201]
NERKI ±	Normal E2 levels, P (twice less)	Mutated allele in DBD (E207A/G208A, or AA) domain introduced onto the ERα-KO background, also described as mouse with ERE-independent signaling	Normal mammary development with decreased side branching and impairment of alveolar bud formation	[210]
ERα-EAAE (DBD)		Four aminoacid exchange (Y201E, K210A, K214A, R215E) on DNA-binding domain (DBD)	Rudimentary mammary gland development	[209]
ENERKI (LBD)		Mutation of G525L on the LBD of ERα	Rudimentary mammary gland development	[211]
ERα-AF1°	Two fold increase of E2	Deletion of aa 2–148 of A/B domain, deleting the AF1 transactivation function	Absence of pubertal development and alveologenesis following transplantation into WT stroma	[62, 138]
AF2ERKI		L543A, L544A point mutations in helix 12, deleting the AF2 function	Rudimentary mammary gland similar to ERα-KO mice	[207]
ERα-AF2°	4–ten fold increase of E2	Deletion of AF2 domain (aa 543–549)	Absence of pubertal development and alveologenesis following transplantation into WT stroma	[138, 208]
MOER(Membrane-Only- Estrogen Receptor)	Elevated E2 levels (5X)	Mouse expressing only a membrane E domain of ERα due to insertion of 20 aminoacid sequence of the neuromodulin protein, which is palmitoylated	Absence of pubertal development, similar to ERα KO mice	[214]
NOER (Nuclear Only Estrogen Receptor)	Elevated E2 levels, decreased P levels	Mutation of palmitoylation site C451 into Alanine of ERα	Profoundly diminished side branching in adult virgin females	[115]
C451A-ERα		Mutation of palmitoylation site C451 into Alanine of ERα	Delayed mammary gland at puberty, and decreased side branching in virgin mice. Basal cells transplanted into WT stroma failed to grow except if reconstituted with ERα-positive luminal cells	[114, 212]
DPM mice		Overexpression of the Disrupting Peptide Mouse (DPM) (aa 176–253) to inhibit ERα interaction with striatin	Not determined	[117]
MMTV-ERα36		ERα36 cDNA expressed under the control of the MMTV promoter	Invasion of mammary fat pad after the puberty with alterations such as stromal thickening, and epithelium thinning	[215]
Pharmacological tools to activate specific functions of ERα				
PaPEs		Specific activation of membrane ERα	Absence of stimulation of mammary gland development	[119]

The first studies were conducted using two distinct knockout mice termed ERαNeoKO [11, 198] and ERα-KO [199]. The mammary epithelial tree of these mice was normally developed at the prepubertal stage indicating that the early stages of mammary morphogenesis are independent of ERα signaling. In contrast, TEB formation and ductal growth were abrogated in the pubescent mutant females [11, 12]. Of note, the ERα-KO mouse completely lacks ERα transcript expression, whereas the ERαNeoKO was found later to retain a substantial ERα function, by producing a spliced mRNA that gives rise to a receptor lacking part of the ligand-independent AF-1 domain, a form reminiscent of that from the ERα-AF1° deficient mice [62, 200].

As ERα-KO mice presented endocrine abnormalities that could indirectly impact their mammary phenotype, orthotopic transplantation assays were performed. This strategy allows to compare the development of wild-type (WT) and mutant mammary epithelial fragments grafted into cleared contralateral mammary fat pads of a WT recipient mouse and thereby reveal mammary epithelium intrinsic phenotype [3]. Unlike WT, ERα-KO ducts grafted into a WT stroma completely failed to develop, even after a hormonal stimulation of the host mouse mimicking pregnancy, demonstrating that ERα expression in epithelial cells is essential for ductal and alveolar development [12].

The importance of epithelial ERα expression for pubertal mammary gland development was further confirmed using a Cre-Lox-based conditional knockout model (MMTV-Cre-ERα^fl/fl^) targeting all luminal cells [201]. This work also included the analysis of the mammary phenotype of WAP-Cre-ERα^fl/fl^ females, a model in which ERα was deleted from luminal cells at late pregnancy and during lactation. Although nuclear ERα is absent from WAP-expressing milk secretory cells, lobuloalveolar development and milk production were perturbed in the WAP-Cre-ERα^fl/fl^ females upon successive pregnancies. Conceivably, the maintenance of early alveolar progenitors, potentially analogous to the so-called parity-identified mammary epithelial cells that express WAP and survive involution might be affected by ERα loss either directly or indirectly [202].

Importantly, transplantation assays using a mix of WT and ERα-KO epithelial cells indicated that ERα in epithelial cells acts in a paracrine manner on neighbor cells [12]. Activation of ERα by E2 was found to induce, in addition to PR expression, the secretion of amphiregulin (AREG) in the epithelium [190]. AREG was the most abundant EGF-like growth factor in the pubertal mammary gland, with a maximum expression 12 h after E2 stimulation in ovariectomized mice. Analysis of AREG-KO and mix WT-KO mammary grafts showed that AREG acts as an essential paracrine mediator of ERα signaling and is required for the massive epithelial cell proliferation, TEB formation and ductal elongation during puberty. Nonetheless, AREG-KO mammary grafts, although poorly developed, expressed PR and consistently could undergo side branching and alveologenesis in a pregnant host, whereas ERα-KO were unable to do so [12, 190].

Additional studies showed that the transmembrane form of AREG is cleaved into a mature peptide by metalloproteinase domain containing protein 17 (ADAM17) and promotes signaling in stromal cells through binding to EGFR [203]. EGFR activation induces expression of growth factors in stromal cells, in particular members of the FGF family that regulate mammary epithelial growth in a paracrine fashion [130]. ERα, AREG and EGFR knockout mice display similar mammary phenotypes, characterized by a lack of ductal development [204–206].

A rudimentary mammary gland similar to the one observed in ERα-KO mice was observed in the ERα-AF2KI mice, in which L543A and L544A point mutations in helix 12 were introduced, deleting the AF2 function [207]. More recently, the roles of AF1 and AF2 transactivation functions of ERα have been explored independently, using the ERα-AF1° and the ERα-AF2° mice generated by P. Chambon and colleagues, respectively [62, 138, 208]. This second ERα-AF2° mouse model was obtained by deleting the aminoacids 543 to 549 in the helix 12 [208]. The data showed that deletion of AF-1 or AF-2 blocks pubertal ductal growth and alveologenesis and by means of grafting assays, revealed an unexpected complexity of ERα signaling, linked to cell-population-specific functions of AF1 and AF2. ERα^high^ luminal cells were found to require both AF-1 and AF-2 to transcribe crucial downstream effector genes such as *Areg*, *Pgr, Prlr* and *Wnt4*. On the other hand, ERα^low^ luminal cells appeared essential for ductal development during puberty but growth inhibitory during pregnancy. This population depends on the AF2 transcriptional response that also controls transcript levels of genes linked to cell motility, adhesion and plasticity [138].

Two mouse models have been mutated into the DNA-binding domain to dissect DNA-binding-dependent vs. ERE-independent transcriptional regulation elicited by ERα: first, the ERα-EAAE (ENERKI) mouse harboring four aminoacid exchange (Y201E, K210A, K214A, R215E) on DNA-binding domain (DBD) [209], and second, the NERKI mouse mutated into the P box of the first zinc finger of the DBD (E207A/G208A)[210]. While results observed with the NERKI mouse bred onto the ERα-KO mice can be questioned due to an unclear figure even in WT, the ERα-EAAE clearly shows a rudimentary mammary gland development [209] similar to that observed in ERα-KO, demonstrating the importance of the DNA-binding nuclear response.

In another model, a specific point mutation (G525L) was introduced on the ERα ligand-binding domain (LBD) to distinguish ligand-induced and ligand-independent ERα actions. This model confirmed that estrogen-induced activation of ERα is crucial for the development of female reproductive tract and mammary gland [211].

As detailed in Sect. 1.2 (Fig. 2), ERα outside the nucleus can activate rapid/non-genomic/membrane-initiated steroid signals (MISS). To analyze the potential implication of MISS in tissue development, two groups have generated similar knock-in mouse models mutated for the palmitoylation site (theoretically the same point mutation), i.e., the ERα-C451A [114] and the NOER mice [115]. In contrast to mice deleted for nuclear effects of ERα, NOER and ERα-C451A mice have a developed mammary gland that completely filled the fat pad but showed diminished ductal side branching and decreased number of blunted duct termini [115, 212].

The specific mechanisms that control the ability of basal and luminal cells to respond to membrane ERα signaling have been investigated in details using the ERα-C451A mouse model and grafting assays [212]. The data demonstrated that mutation of the palmitoylation site of ERα was necessary in promoting intercellular communications essential for mammary gland development. In fact, absence of the membrane ERα impairs the expansion of ERα positive luminal cells that further alters the required paracrine signaling and the final ductal outgrowth. Transcriptional analysis also points the requirement of *Greb-*1 gene expression. *Greb-1* is well-known as an early response gene in the ERα-regulated pathway and was shown to be a chromatin-bound ER coactivator essential for ER-mediated transcription that stabilizes interactions between ER and additional cofactors [89, 213]. Importantly, loss of membrane signaling in luminal cells also altered *Jak2* and *Stat5a* gene expression, a pathway found at the crossroad of hormonal and growth factor signaling which uncovers an important role of membrane ERα as a key regulator of growth factor response [212].

A transgenic mouse deprived of both nuclear and cytoplasmic functions of ERα was also developed by expressing only a functional E domain of ERα at the plasma membrane in an ERα-KO background to study the specific membrane actions of ERα [214]. This MOER mouse harbors a rudimentary mammary gland development similar to the ERα-KO mice. The absence of pubertal mammary ductal growth following activation of only membrane actions of ERα was also confirmed using a pharmacological tool, the “pathway preferential estrogens” (PaPEs). These ligands were synthesized to preserve their essential chemical and physical features to bind ERα with an affinity that allowed preferential induction of the extranuclear-initiated signaling/MISS pathway. PaPEs did not stimulate mammary gland fat pad filling nor breast cancer cells growth [119].

Finally, to investigate the potential function of the short ERα-36 isoform, only present in humans, a MMTV-ERα36 transgenic mouse strain was generated allowing specific expression of ERα36 cDNA in mammary epithelial cells. The mammary epithelium of the mutant females normally invaded the fat pad but significant defects were observed, such as duct dilation, stromal thickening, epithelium thinning and leakage [215].

Collectively, the data obtained from mouse models revealed the complex status of ERα expression in the mammary epithelium and the multiple implications of ERα signaling in the control of mammary development. Genomic actions include induction of crucial paracrine effectors such as AREG required for ductal growth and of PR expression necessary for the expansion of the secretory tissue. Both AF-1 and AF-2 genomic actions of ERα are crucial for a normal mammary development during puberty and pregnancy. In addition, non-genomic effects of ERα signaling that modulate intercellular communications participate in the regulation of mammary morphogenesis. It is possible that the levels of circulating estrogens, lower in puberty than during pregnancy, direct and trigger differential ERα responses in estrogen-sensing cells.

### Mouse models mutated for pioneer factors and coregulators of ERα

As previously mentioned, the transcriptional activity of ERα depends on its interaction with coregulators. Consistently, several of these coregulators appeared to be critical for TEB formation, ductal branching and alveologenesis during mammary gland development (reviewed in [181]).

FOXA1 was the first pioneer factor identified for ERα, specifically required for ERα induced transcription of cyclin D1 [216]. Co-expression of FOXA1 and ERα was observed not only in the pubertal gland [150] but also in luminal breast cancers and cell lines [217–219]. The crucial role of FOXA1 in mammary morphogenesis was confirmed using orthotopic and renal capsule transplantation of mammary anlagen from *Foxa1* KO mice [150]. These assays revealed that ductal elongation and TEB formation were severely impaired in the absence of FOXA1, whereas alveologenesis, although limited, could occur in pregnant hosts. IHC studies showed that FOXA1-deficient luminal cells lacked ERα and PR expression, whereas FOXA1 was expressed in ERα-KO mammary gland, indicating that FOXA1 acts upstream of ERα and controls its expression and signaling.

Similar to ERα and FOXA1, GATA3 is required for TEB formation and ductal growth during puberty. Accordingly, the targeted loss of GATA3 in the mammary epithelium leads to a defective generation of ductal ERα-expressing cells, an accumulation of ERα-negative luminal progenitors and a block in differentiation, revealing a pivotal role for GATA3 in the maintenance of the luminal compartment [182, 220, 221].

Collectively, these studies revealed a complex interplay between ERα, GATA3 and FOXA1 [181]. GATA3 regulates FOXA1, which in turn regulates ERα, while GATA-3 and ERα regulate each other positively. Furthermore, these factors colocalize at transcription sites upon E2 stimulation and form a tripartite complex that ensures optimal transcriptional activation [183, 222–224]. ERα also upregulates FOXM1, another forkhead transcription factor that down-regulates GATA3 expression and may balance ERα and GATA3 interaction during mammary gland development. FOXM1 was found to promote luminal cell proliferation as opposed to GATA3 that mediated luminal differentiation [225]. The chromatin complex formed by ESR1, GATA3, and FOXA1 thus coordinately orchestrates mammary luminal lineage commitment and estrogen response.

More recently, ten–eleven translocation (TET2), a chromatin modifier which mediates DNA demethylation, was found highly expressed in mammary luminal cells [226]. Targeted deletion of TET2 in the mammary epithelium through MMTV-Cre showed that loss of TET2 increased ductal branching and TEB numbers in pubescent females but impaired alveolar development at pregnancy. FACS analysis of the mutant glands revealed an increased proportion of mammary basal cells with stem cell activity, a diminished subset of ERα ^+^ luminal cells and an aberrant commitment of luminal cells towards a mixed basal/luminal phenotype. TET2 was found to interact with the transcription factor FOXP1 and forms a chromatin complex that mediates demethylation of *Esr1, FoxA1* and *Gata3.* TET2 loss led to a decreased expression of ERα, FOXA1 and GATA3 expression both at protein and mRNA levels that profoundly perturbed the luminal lineage commitment and the balance between the basal and the luminal lineages and thereby altered mammary development.

Among the main co-activators of ERα are also members of the p160 family (SRC-1, SRC-2 and SRC-3), as mentioned in Sect. 1. SRC-1 disruption in vivo showed decreased mammary ductal branching and also decreased number and size of alveoli during pregnancy, even though milk production was normal [227]. In contrast, SRC-2 is not required for early post-natal mammary gland development, in both virgin and pregnant mice [228]. However, work from Mukherjee and colleagues [229] reported that SRC-2 may be important for progesterone-induced signaling. As SRC-2, SRC-3 is not essential for E2-stimulated ductal growth in virgin mice, but is required for progesterone-stimulated cellular proliferation and glandular differentiation during pregnancy [230]. In summary, SRC-1 is an important coregulator of ERα for ductal branching at puberty and SRC-3 is probably the primary coactivator for PR in breast [231].

CITED 1 (Cbp/p300-interacting transactivator with Glu/Asp-rich carboxy-terminal domain) was identified as another important coregulator of ERα controlling the pubertal mammary ductal morphogenesis, as shown by the analysis of CITED1 homozygous null mice [186].

Among the main corepressors, the role of REA in mammary gland development during puberty or pregnancy was, respectively, studied using conditional tissue-specific deletion of one or both alleles of REA under the control of *Pgr* or *Wap* promoter, respectively [232]. Interestingly, at both puberty and pregnancy, opposite effects were observed depending of the homozygous or heterozygous deletion, demonstrating that the REA is crucial for mammary gland development at all stages, puberty, pregnancy and lactation, with crucial gene dosage-dependent actions. *Rip140*-deficient mice and transgenic *Rip140* overexpressing mice have also been generated [233]. The *Rip140* KO mice displayed minimal ductal branching at maturity. In contrast, the ductal network of the *Rip140* overexpressing mice was more branched, exhibited hyperplasic growth and developed denser alveolar structures. In fact, RIP140 expression is essential in both the epithelium and the stroma and acts as a rate-limiting factor required for ductal development in the mammary epithelium. RIP140 acts as a coregulator of ERα and is recruited to a number of its target gene promoters/enhancers, such as *Areg, Pgr*, *Ccnd1* and *Stat5a.*

### Estrogens acts in concert with other growth factors

Numerous data have demonstrated that estrogens act in concert with growth factors and the cooperation between estrogens and growth hormone (GH) in governing pubertal development has been particularly studied. The main downstream effector of pituitary-derived GH signaling is IGF-1 (insulin growth factor 1) primarily produced by liver but also locally by mammary stromal cells [3, 130]. The *Igf1*-KO mice have an impairment of mammary development and lack TEBs, a phenotype that cannot be restored by the injection of estrogen while the injection of IGF-1 alone for 5 days improves development [234, 235]. The receptor involved in this signaling was investigated using embryonic *IGF-IR* mammary gland transplantation into WT stroma, because null mice die at birth. These data directly demonstrated that IGF-IR expressed by TEB cells is necessary for proliferation and ductal morphogenesis [236]. In contrast, these defects are corrected during pregnancy, indicating that exposure to signals from pregnancy is able to compensate for the loss of otherwise important mammary signaling pathways. This restoration during pregnancy may also result from changes in mammary cell sensitivity to insulin-like signals mediated by the Insulin receptors (IR). Indeed, there is genetic evidence that the IR can mediate the growth promoting function of IGF-2 [237], that was also confirmed by showing that IGF-2 was a downstream mediator of prolactin-induced alveologenesis and an upstream regulator of cyclin D1 expression [238]. IGF-1 may play a crucial role during post-natal development in concert with ERα while IGF-2 might drive the prolactin effect during alveologenesis. Moreover, overexpression of IGF1R in epithelial cells in mice leads to abnormal development of the ducts (hyperplasia) and tumor formation in vivo [239].

Tian and colleagues have particularly studied ERα/IGF1R co-signaling using a mouse model overexpressing human IGF1 in the mammary gland under the control of the basal-specific bK5 promoter [240]. This ectopic IGF-1 expression in myoepithelial cells induced paracrine effects on adjacent epithelial cells. Interestingly, this study shows that ectopic IGF-1 is able to activate different signaling pathways dependent on the pubertal status of mice. Indeed, the results show an increase in p-Akt associated with the activation of mTOR in the prepubertal transgenic glands whereas in the post-pubescent transgenic glands, the activated pathways are related to the Ras/Raf/MAPK signaling cascade. These observations can be correlated with the change in the expression of ERα in the mammary gland. ERα is more expressed in the pubescent gland than in the post-pubescent gland, which corresponds to negative feedback by the E2 ligand. It is then proposed that IGF1/IGF1R/ERα signaling may activate different cytoplasmic effectors depending on the proliferative state of the mammary gland.

## Estrogens and breast cancers

### ERα-positive luminal breast cancers

Considerable interest has focused on luminal cells in the context of mammary gland development and tumorigenesis, as most breast cancers are thought to originate from deregulated luminal cells, either negative or positive for ERα [4, 241]. ERα-positive tumors account for 70–80% of all breast cancers and belong to the two luminal molecular subtypes, A and B, characterized by a low and high proliferation index, respectively [13–15]. The most frequent special histological subtype is the invasive lobular carcinoma (ILC) that clusters with luminal A and B subtypes and is characterized by a loss of E-cadherin expression [15].

Most ERα-positive breast cancers depend on estrogen for their growth and ERα expression is predictive for responsiveness to endocrine therapies targeting the E2/ERα pathway. It is important to mention that histologically, ERα-positive tumors are defined as having at least 1% of tumor cells exhibiting a nuclear ERα staining as assessed by IHC, without a clear consensus of the used antibodies [14, 15]. Hence, ERα-positive tumors are highly heterogeneous with a broad range of ERα expression spanning from 1% to nearly 100%. In addition, they display an important intratumoral heterogeneity, as highlighted by a recent work using imaging mass cytometry at the single cell level [242, 243].

Blockade of E2/ERα activity by administration of tamoxifen and aromatase inhibitors have major antitumor effects on ERα-positive breast cancers and already benefited to millions of women [244]. This benefit is still observed when only a small fraction of breast cancer cells expresses ERα, demonstrating the importance of blocking the expansion of this cell subset and its potential paracrine action. Nonetheless, late relapses at distant sites are often observed, compromising the long-term outcome of patients with ERα-positive breast cancers. In addition, an important proportion of the patients do not respond to endocrine therapies and up to 50% acquire resistance under treatment [245].

### Exposure to estrogens and breast cancers

The impact of estrogens on breast cancer was first demonstrated more than a century ago by the British surgeon George Beatson who observed regression of a breast tumor following ovariectomy [246]. Nowadays, early and prolonged exposure to endogenous or exogenous estrogens during a woman's life is recognized as being a factor of major risk in developing a breast cancer, in particular an ERα-positive subtype [247, 248]. Early menarche, late menopause, nulliparity or late first pregnancy are viewed as risk factors while breast feeding is considered as a protective factor [247, 249, 250]. The timing of hormone exposure appeared as an important parameter since aberrant hormonal exposure prior to puberty or in early life has a more significant effect on breast cancer risk than late menopause, suggesting a particular susceptibility of the immature mammary gland to tumorigenesis [247].

The risk of breast cancer also increases among women who currently or recently used contemporary hormonal contraceptives as compared to non-users. This absolute increase in risk remains low but rises with longer durations of use [248, 251]. According to the big prospective Women Heath’s Initiative (WHI) trial that evaluated risks of hormonal replacement therapy, the combination of conjugated equine estrogens plus medroxyprogesterone acetate led to an increased risk of breast cancer whereas hysterectomized women treated with estrogens alone (equine conjugates, without progestin) developed, quite unexpectedly, less breast cancer than women receiving a placebo [ 252, 253]. More recent analyses have shown that the levels of risks varied between types of hormonal replacement therapies, with higher risks when progestins were used in the combination with estrogens (as compared to the natural progesterone), and again, for longer duration of use [254]. The identification of safer estrogenic compounds is, therefore, necessary to improve the benefit / risk balance in patients on hormonal replacement therapies and contraception.

### Mutations of ESR1 in human breast tumors

The most frequent mutated genes in ERα-positive breast cancers are *PIK3CA*, *GATA3*, *MAP3K1*, *KMT2C* and *TP53*. Mutation of CDH1 (encoding E-cadherin) or loss of alleles are common in the lobular subtype (reviewed in [15, 255]). In contrast, ESR1 mutations are rare (less < 1%) in primary ERα-positive breast cancers [256] but between 20 and 40% of ESR1 mutations are observed in metastatic breast cancer and influence response to hormone therapy (reviewed in [256–260]). In fact, these mutations emerge under the pressure of chemotherapy and successive anti-hormonal treatments, often after aromatase inhibitor (AI) treatment. They include highly recurrent ESR1 mutations encoding Y537C/S/N with a prevalence reaching 60% of mutations detected in metastatic breast cancers [261, 262]. Another mutation in the LBD is the D538G, at a high frequency of 20% [260]. This mutated tyrosine Y537 has been particularly involved in the growth of mammary cancer cells and xenografts following phosphorylation by Src tyrosine kinases (p56^lck^ and p60^c−src^) [263–267]. In addition, mammary MCF7 cancer cell lines stably expressing the ERα-Y537S/N and D538G present higher proliferation than wild-type expressing cells. Moreover, these mutations not only confer constitutive, hormone-independent activity of ERα but also lead to change in transcriptional responses that mediate cancer progression and confer anti-estrogen resistance by altering the conformation of the ligand-binding domain of ERα, which leads to a stabilized agonist state and an altered antagonist state [268, 269]. Expression of the ESR1Y537S mutation also induced an epithelial–mesenchymal transition (EMT) in cells and exhibited enhanced migration [270]. Other mutations, such as K303R, E380Q, S463P, V534E, Y535S, L536R were also found with different frequencies [271–275]. A summary of the characteristics of all these ERα mutants in breast cancers have been recently reviewed in [276].

More recently, genomic rearrangement events producing *ESR1* fusion genes have been reported in endocrine therapy resistance [277]. These events include in-frame fusions such as inter-chromosomal ESR1 translocations with the YAP1 gene (ESR1-e6 > YAP1), the protocadherin 11 X-linked gene, PCDH11X (ESR1-e6 > PCDH11X) and the nucleolar protein 2 homolog gene, NOP2 (ESR1-e6 > NOP2), and 2 intra-chromosomal translocations with the A-kinase anchoring protein 12 gene, AKAP12 (ESR1-e6 > AKAP12) and the DNA polymerase eta gene, POLH (ESR1-e7 > POLH). These ESR1 fusion genes not only led to endocrine resistance but also induced epithelial–mesenchymal transition (EMT) leading to metastasis.

Finally, there are also numerous changes in the chromatin landscape and epigenetic mechanisms regulating the biology of ERα-positive breast cancer that can orchestrate the resistance to breast cancer treatments (reviewed in [278]).

### Models of ERα-positive breast cancers

Establishing in vivo models mimicking the complex biology of ERα-positive breast cancers remains an active field of research (reviewed in [279]. Since the 1980s, different approaches have been used including chemically induced carcinomas in rats, genetically engineered mouse models (GEMMs), human cell line xenografts and patient derived xenografts (PDX), each having their own advantages and limitations.

GEMMs have contributed significantly to the field of breast cancer research and translational oncology, however, most of them develop ERα-negative mammary tumors [280]. Nonetheless, the broadly used MMTV-PyMT mouse model that expresses polyoma middle T (PyMT) oncogenic protein in the mammary epithelium recapitulates some aspects of ERα-positive breast cancers. This model rapidly develops spontaneous luminal-like ERα-positive premalignant mammary lesions, sensitive to tamoxifen, which further progress to ERα-negative mammary carcinoma forming lung metastases [281, 282]. In MMTV-PyMT females, ERα signaling favors tumor onset, tumor growth and pulmonary metastasis [282]. Loss of TET2 that profoundly alters ERα signaling and mammary development was recently found to promote the growth of invasive MMTV-PyMT tumors and confer resistance to tamoxifen in vivo [226].

A conditional tetracycline-responsive transgenic mouse model overexpressing ERα in mammary epithelial cells was generated that developed proliferative lesions such as atypical ductal and lobular hyperplasia and ERα^ +^ PR ^+ ^ductal carcinoma in situ by 4 weeks of age [283]. Moreover, a transgenic mouse expressing the mutation found in human tumors was created by expressing the HA tagged K303R-ERα under the control of the MMTV promoter [284]. Although more alveolar budding was observed in 4-month-old mutant K303R-ERα transgenic mice as compared to WT-ERα-MMTV mice, no hyperplasia was observed in older mice.

Among recent GEMMs of interest are the Stat1-null and the BlgCre; KiRas^(G12V)^ mice. Stat1-null females spontaneously develop mammary adenocarcinomas of luminal origin that comprise more than 90% ERα^+^ and PR^+^ cells and depend on estrogen for tumor engraftment and progression. Although accelerated by parity, the tumor latency of about 10 months hampers an easy use of this model [285]. Conditional expression of the mutated human KiRas under the control of the *Blg* promoter, active during pregnancy and lactation, leads to the development of invasive ductal carcinomas within 3–9 months after induction. These tumors, positive for ERα and PR but negative for HER2, mimic the luminal A subtype and respond to anti-estrogen treatment [286].

As PI3KCA mutations are commonly found in luminal breast cancer subtypes, two groups used inducible GEMMs to investigate the impact of an oncogenic PI3KCA mutant targeted either in basal or luminal cells and analyze its contribution to tumor heterogeneity [287, 288]. Interestingly, PI3KCA mutant expression in basal cells induced the formation of luminal ERα^ +^ PR^ + ^mammary tumors while its expression in the whole luminal population gave rise to luminal ERα^ +^ mammary tumors and basal-like ERα^-^ PR^-^ tumors. Thus, the same mutation can induce plasticity in normally lineage-restricted cell types and result in different tumor phenotypes, reinforcing the importance of the cell of origin in breast cancer development [4]. The use of specific promoters for addressing pertinent oncogenic mutations in the ERα ^+^ luminal cell lineage should lead to the design of novel GEMMs, providing further insights into initiation and progression of the ERα^ +^ luminal breast cancers.

Finally, many PDX models have been successfully established for pre-clinical breast cancer research, however, the take rates of ERα-positive tumor samples transplanted in the mammary fat pad of immunocompromised mice were noticeably low [289, 290]. Recently, intraductal grafting has enabled the establishment of ERα-positive PDX derived from fresh human tumor biopsies with significantly improved take rates [291, 292]. These PDX models that recapitulate early developmental stages of ERα-positive luminal breast cancers should be of great help to evaluate aggressiveness and responsiveness to endocrine therapy. The same strategy was further used to design a model of ERα-positive ILC and test novel therapeutic approaches [293]. Gene expression analysis of the ILC-derived samples revealed an ECM remodeling signature with an enrichment in LOXL1, a targetable member of the lysyl oxidase family. LOXL1 inhibition through a pan LOX inhibitor was found to reduce tumor growth and metastasis by human lobular cell lines injected intraductally.

## Conclusion

Since the cloning of *ESR1* in 1986, the field has made considerable advances in deciphering the molecular mechanisms of ERα signaling through genomic and non-genomic actions and in addition, piecing together the role of ERα in luminal cells and in mammary gland development and function. These advances largely rely on the recent technological developments, including sophisticated transgenic mouse models, high-throughput sequencing and advanced confocal microscopy.

Analysis of the mammary phenotype from multiple transgenic mouse models, targeting ERα or its main coregulators, has clearly shown that ERα signaling does not play a role before puberty, whereas it is essential for pubertal ductal growth and subsequent alveologenesis. In the last decade, the ERα-expressing luminal cell lineage has been better characterized in terms of ERα transcript and protein levels, molecular profiles, stem/progenitor cell content, proliferation ability and differentiation potential. The crucial role of ERα-expressing luminal cells in sensing hormonal stimuli and sending paracrine signals to their neighbors, the basal and ERα-negative luminal cells, has been confirmed and refined. These signals control the amplification of the ductal cells during puberty and the expansion of the secretory tissue during gestation.

At the mechanistic level, significant progresses have been made in deciphering the role of ligand-independent and ligand-dependent activation functions of ERα. In particular, it has been shown that AF1 and AF2 domains have cell population-specific functions but are both required for a proper expression of paracrine mediators [138]. The target cells of the non-genomic membrane actions of ERα signaling within the mammary epithelium remain to be precisely identified. However, data from transgenic mouse models revealed that this non-classical mode of action, active for example in endothelial cells, participates in the control of mammary development by regulating intercellular communication [212]. Similarly, classical and non-classical progesterone signaling pathways through nuclear and membrane receptors have been identified in mammary epithelial and cancer cells [153].

Genomic and non-genomic ERα signaling likely act in concert according to the developmental stage of the mammary gland, its hormonal context and the differential levels of circulating estrogens. Undoubtedly, a better understanding of this complex interplay will shed more light on the control of the mammary basal and luminal cell lineages and their deregulation during the tumorigenic process.

The upstream regulation of ERα ^+^ expression in the mammary epithelium is less understood than its action. An important direction for future research is to further define the niche of ERα ^+^ luminal cells and identify niche signals regulating the development and homeostasis of this lineage. In the mammary ducts, luminal ERα-positive cells directly interact with luminal ERα-negative and basal cells that display-specific cell–cell contacts and secrete multiple growth factors able to specifically impact ERα ^+^ cell function in a juxtacrine or paracrine manner. In addition, luminal ERα ^+^ cells can interact with resident intra-epithelial macrophages, a population lying between the luminal and basal cell layers that was recently revealed using high-resolution imaging [294].

Deciphering the complexity of the mammary stroma (fibroblasts, adipocytes, immune cells) and analyzing its interplay with the epithelial compartment during normal development and tumorigenesis also define a broad research area [243]. Numerous mammary stromal cells express ERα and, therefore, respond to estrogen stimulation.

Finally, it is worth mentioning that several emerging topics could not be developed in the present review, such as chromatin landscape, epigenetic regulation and non-coding RNAs. They all are actively investigated in the context of normal mammary development and breast cancers [4, 5]. Collectively, these efforts should provide a better understanding on how the normal mammary tissue develops and evolves in the course of a woman life, and how the developmental programming is lost during breast cancer initiation.

## Data Availability

Not applicable.
